# Current nursing practice for patients on oral chemotherapy: a multicenter survey in Japan

**DOI:** 10.1186/1756-0500-7-259

**Published:** 2014-04-23

**Authors:** Hiroko Komatsu, Kaori Yagasaki, Kimio Yoshimura

**Affiliations:** 1 Faculty of Nursing and Medical Care, Keio University, 35 Shinanomachi, Shinjuku-ku, Tokyo 160-8582, Japan; 2 Department of Health Policy and Management, Keio University School of Medicine, 35 Shinanomachi, Shinjuku-ku, Tokyo 160-8582, Japan

**Keywords:** Oral chemotherapy, Medication adherence, Compliance, Interdisciplinary care, Medication management

## Abstract

**Background:**

With a paradigm shift toward a chronic care model in cancer, the issue of adherence is becoming increasingly important in oncology.

**Methods:**

We mailed two self-reported surveys on current nursing practices for patients on oral chemotherapy to all 309 designated cancer centers and 141 large general hospitals in Japan. The first survey was based on a nurse-based questionnaire containing 40 items concerning nurse’s characteristics, nurse staffing at workplace, general nursing care for new patients on oral chemotherapy and those with refilled prescriptions, follow-up, and system-based approach. The second survey was based on a patient-based questionnaire containing 10 items about patient characteristics and adherence-related nursing practice for 249 patients taking oral chemotherapy of 903 systematically sampled. We used multivariate logistic regression to identify factors that were associated with adherence-related nursing practices.

**Results:**

A total of 62 nurses (mean age: 41.5 years) from 62 hospitals who consented participated in the both nurse-based survey and patient-based survey about 249 patients. The results of nurse-based survey indicated that practices varied, but nurses were less likely to ask adherence-related questions of patients with refilled prescriptions than of new patients. The results of patient-based survey found that questions on side effects, discussions about barriers to achieving balance between treatment and daily life activities, and medication management were all significantly related to the question about unused medicines. Logistic regression revealed that adherence-related nursing practices were associated with the nurse’s background, type of treatment, and healthcare system-related factors. Patient orientation on oral chemotherapy, interdisciplinary learning, and having a system-based approach for detecting prescription errors were identified as healthcare system-related factors.

**Conclusions:**

A more systematic approach must be developed to ensure patients receive safe and effective oral chemotherapy, while nurses should play significant roles in patient education and monitoring.

## Background

Recent progress in treatment of cancer has accelerated an expansion of the development of oral anticancer agents [1]. The availability of oral anticancer agents has a major impact on cancer care. In oral chemotherapy, some of the traditional responsibilities of healthcare providers have shifted to patients [2]. Despite increased patient’s responsibility for self-management, oral therapy is preferred by patients mainly because of no need for clinic visits, no needle placement, and reduced cost [3-5].

As a growing number of patients are choosing oral chemotherapy over intravenous administration [6], the issue of adherence is becoming increasingly important in oncology [7-9]. Traditionally, adherence was not a major problem in cancer patients compared to other chronic disease [10] because cancer treatment were usually provided in acute care settings, and intravenous chemotherapy has been strictly controlled at outpatient clinic by healthcare providers. The convenience of oral medication has a flip side—serious complications and fatal outcomes. Oral chemotherapy is cost effective, if it is taken as prescribed [11]. However, if patients are non-adherent to medication, the cost burden of unused medicines is substantial.

The terms “adherence” and “compliance” are used interchaneably, but “adherence” is generally preferred to “compliance” because “compliance” suggests that patient is a passive follower of the doctor’s orders, and “adherence” implies that the treatment plan is based on a therapeutic alliance between the patient and the healthcare provider [12]. Optimal adherence is achieved “if no doses are missed, no extra doses are taken, and no doses are taken in the wrong quantity or at the wrong time” [10]. In general, a patient is considered to be adherent if he or she has taken 80% of a prescribed medication [1].

A number of factors are interrelated with medication adherence. There are three levels of barriers to adherence: patient, healthcare provider, and healthcare system levels [12]. The patient-level factors include health beliefs and socioeconomic factors, while communication and the complexity of the regimen are regarded as healthcare-provider factors [8]. Healthcare system issues include duration of treatment follow-up visits, prescribing errors, and conflicting information regarding the consequences of non-adherence [3].

To overcome barriers to patient adherence, there is no single standard intervention [13]. However, multidisciplinary and multimodal strategies are considered as effective [1]. Also, the literature places a primary emphasis on patient education [4,8,14,15]. Education should be tailored for individual patients, and patients generally prefer direct interaction with healthcare providers [4]. Other suggested interventions include systematic monitoring of patient pill taking, and use of pill diaries and pill counting [10].

The primary roles of oncology nurses in patients on oral chemotherapy include patient education, communication, symptom management, and proactive follow-up [16]. Their skills help patients with side-effect management and handling medications at home, and patient adherence to medication can be improved by follow-up care [5]. The ASCO/ONS chemotherapy administration safety standards including oral agents have been established in 2013 [7]. A national web-based survey on current nursing practice patterns for oral chemotherapy in the United States reported that only half of the nurses worked in practices with specific policies, procedures, and resources for patients on oral chemotherapy, and found erratic procedures and inadequate interdisciplinary communication in many practices [17]. Additional research is needed to determine the best practice in patient education, monitoring, and safety management, and furthermore, to identify the gaps that may exist between the nurses’ roles and practice [16,17].

We conducted a multicenter cross-sectional questionnaire survey in designated cancer centers and at large general hospitals in Japan. The goal was to determine a baseline of nursing practices for oral chemotherapy in order to improve medication adherence.

## Methods

### Design

A cross-sectional questionnaire survey was designed and disseminated to 309 cancer centers designated by the Ministry of Health, Labour and Welfare, and 141 general hospitals with more than 300 beds in Japan.

### Participants and procedures

Designated cancer centers were identified using the list of healthcare institutions in 2012 from the Ministry of Health, Labour and Welfare. In November 2012, surveys were mailed to all 309 designated cancer centers as well as to 141 general hospitals that were geographically stratified and randomly selected from all general hospitals with more than 300 beds in Japan. Using systematic sampling, every 10th patient was selected from a list of patients who had visited the chemotherapy clinic in participating hospitals during the week before the survey.

One nurse, who met the following inclusion criteria, was selected from each hospital: (1) more than three years of experience in chemotherapy clinic; (2) experience as a nursing leader in a chemotherapy clinic; and (3) willing to participate in the study.

An initial invitation to take part in the survey was sent to the nursing directors of 450 hospitals. Those who did not reply received phone calls reminding them about the survey; those who elected not to participate were questioned about their reasons. Those who agreed to participate completed the questionnaires on oral and intravenous chemotherapy.

### Questionnaire

The study consists of two surveys: nurse-based and patient-based questionnaires. The nurse-based questionnaire developed for this study was based on a previous study [18] and literature review [4,16,19,20] to determine nursing practice in patients on oral chemotherapy. It consists of a total of 40 items concerning nurse’s characteristics such as age, experience of nursing and oral chemotherapy (8 items), nurse staffing at workplace (4 items), general nursing care for patients who started oral chemotherapy and those with refilled prescriptions including follow-up (20 items), and details of system-based approach such as interdisciplinary learning opportunities and prescription error prevention, and prescription error prevention (8 items). Regarding general nursing care and system-based approach, the respondents answer questions “Yes” or “No”.

A 10-item patient-based questionnaire includes demographic and clinical characteristics of patient (5 items: age, gender, type of cancer, and primary or recurrent treatment) and “Yes” or “No” questions about adherence-related nursing practice for individual patients (5 items: side effects, balance between treatment and daily life activities, self-management, nurse’s role of monitoring medication management, and unused medicines). Nurses in charge at chemotherapy clinics, who replied to the nurse-based questionnaire, fulfilled this patient-based questionnaire.

### Ethical considerations

The study procedures were approved by the Institutional Review Board at Keio University (Approval Number: No 198, 2012). In addition, the participating hospitals individually obtained study approval if necessary. We obtained informed consent from all participating hospitals, and anonymity of responses was ensured. We obtained consent from the nurses and patients.

### Statistical analysis

To assess differences between the question on unused medicine and nursing practices (addressing side effects, the balance between treatment and daily life, medication management and monitoring), we used Fisher’s exact test. A multivariate logistic regression was used to identify factors associated with adherence-related nursing practices (the question on unused medicine). Explanatory variables were selected for inclusion in the model using the stepwise selection procedure. The significance level was set at 0.05.

## Results

Of the 450 hospitals providing ambulatory chemotherapy, 388 declined to participate in the study. Reasons for non-participation included refusal or non-response of person in charge (the nursing director forwarded the questionnaire to the nurse in the chemotherapy clinic but did not receive a reply) (n = 257), lack of time or staffing (n = 58), prior participation in other surveys (n = 83), practical difficulties (n = 40), and other (n = 66). A total of 62 hospitals consented, and 62 nurses in those hospitals participated in the survey. Of 930 patients who were systematically sampled, 249 had received oral chemotherapy. Therefore, 62 nurses fulfilled the patient-based survey about these 249 patients who those nurses cared for.

Mean age of the nurse respondents was 41.5 (SD 6.4) years. Mean years of nursing and chemotherapy experience were 19.4 (SD 6.4) and 11.6 (SD 5.9), respectively. Most of the nurses (72.6%) were oncology certified nurses working at chemotherapy or outpatient clinics (Table 1).

**Table 1:** Nurse characteristics

	**Mean**	**SD**
**Age**	41.5	6.4
**Years of nursing experience**	19.4	6.4
**Years of chemotherapy experience**	11.6	5.9
**Educational Level**	**n**	**%**
Junior college and nursing school	56	90.3
≧Bachelor’s degree	6	9.7
**Job title**		
Nurse administrator	5	8.1
Senior charge nurse	27	43.5
Staff nurse	30	48.4
**Certification of oncology nurse**		
Yes	45	72.6
No	17	27.4
**Practice setting**		
Chemotherapy clinic	49	79.0
Outpatient department	13	21.0

n = 62.

### Nurse-based survey

Table 2 shows nurses’ perceptions of nursing processes for oral chemotherapy. For new patients receiving oral chemotherapy, more than 90% of nurses reported that they asked their patients about emergency contacts, side effects, and family/friend support, and provided patient education on medication. In contrast, only 30.6% of nurses asked whether patients were confident about managing their medication. Patient understanding (58.1%) and medication schedule (64.5%) were rated lower than expected. Nurses were less likely to ask adherence-related questions of patients with refilled prescriptions than of new patients. Regarding unused doses of anticancer agents, 35.5% of nurses reported that they did not confirm the number of unused doses when patients had refilled prescriptions.

**Table 2:** Nurse-based survey: nurses’ perceptions on nursing processes

	**Yes**		**No**	
	**n**	**%**	**n**	**%**
**New patients on oral chemotherapy**				
**Medication Schedule**				
1. Do you confirm the understanding of new patients by letting them explain their medication schedule to you?	40	64.5	22	35.5
**Education**				
2. If the patient does not fully understand the medication protocol, do you give them information about the medication?	58	93.5	4	6.5
**Side Effects**				
3. Do you check whether the patient understands common side effects?	51	82.3	11	17.7
**Emergency Contact**				
4. Do you check whether the patient knows the emergency contact?	56	90.3	6	9.7
**Management of Side Effects**				
5. Do you talk about practical coping methods for side effects with the patient?	58	93.5	4	6.5
**Barriers to Adherence (Physical)**				
6. Do you ask the patient about physical symptoms (e.g., feeling of numbness in hands/legs) as barriers to taking the medication?	56	90.3	6	9.7
**Patient Confidence in Medication Management**				
7. Do you ask the patient how confident they are about managing their medication?	19	30.6	43	69.4
**Balance Between Treatment and Daily Life Activities**				
8. Do you talk to the patient about potential barriers to achieving balance between treatment and daily activities?	39	62.9	23	37.1
**Support**				
9. Do you offer support to the patient to encourage him/her or to ease anxiety?	58	93.5	4	6.5
**Family/Friend Support**				
10. Do you ask the patient whether he/she has a supportive family/friend?	58	93.5	4	6.5
**Financial Burden**				
11. Do you ask the patient whether he/she feels financially burdened?	43	69.4	19	30.6
**Social Resources**				
12. Do you provide information on social resources to the patient if financial problems are a barrier to treatment?	53	85.5	9	14.5
**Patient Understanding**				
13. Do you ask the patient about his/her understanding of oral chemotherapy (e.g., effectiveness and preference)?	36	58.1	26	41.9
**Patients with Refilled Prescriptions**				
**Patient's Knowledge**				
1. Do you ask the patient about how he/she currently manages his/her medication schedule?	44	71.0	18	29.0
**Skipping**				
2. Do you ask the patient whether he/she ever skips a dose of his/her oral anticancer medication?	55	88.7	7	11.3
**Non-compliance**				
3. Do you ask the patient whether he/she has unused medicines?	40	64.5	22	35.5
**Reasons for Non-compliance**				
4. Do you ask the patient about his/her reasons for not following the physician's instructions?	52	83.9	10	16.1
**Support for Resolution**				
5. Do you talk about problem-solving methods in regards to medication management?	46	74.2	16	25.8
**Report of Skipping to Healthcare Professionals**				
6. Do you ask the patient whether he/she tells his/her healthcare providers when he/she skips a dose of medicine?	48	77.4	14	22.6
**Support for Management of Side Effects**				
7. Do you provide the patient with further advice about coping methods for side effects?	47	75.8	15	24.2

(n=62).

System-based approaches are shown in Table 3. While 40.3% of nurses provided orientation on oral chemotherapy to patients before initiation of therapy, 32.3% of hospitals reported offering interdisciplinary learning opportunities on oral chemotherapy, and 43.5% had a system-based approach for detecting prescription errors of oral anticancer agents. Only one hospital offered a telephone follow-up for patients on oral chemotherapy.

**Table 3:** Nurse-based survey: system-based approach

	**Yes**		**No**	
	**n**	**%**	**n**	**%**
**Patient Orientation**				
1. Do you provide orientation to new patients on oral chemotherapy?	25	40.3	37	59.7
**Telephone Follow-up**				
2. Is it routine to follow up with patients by telephone after they have received their first course of oral chemotherapy?	1	1.6	61	98.4
**Emergency Contact**				
3. Are patients on oral chemotherapy given an emergency contact number (including for nights/holidays)?	55	88.7	7	11.3
**Patient Consultation and Education**				
4. Is there any process in place to provide consultation and education to patients on oral chemotherapy?	28	45.2	34	54.8
**Education for Emergency Nurses**				
5. Does your institution offer learning opportunities for nurses who provide care for patients on oral chemotherapy in the emergency department?	7	11.3	55	88.7
**Collaboration Between Chemotherapy and Emergency Nurses**				
6. Is there any collaboration system in place for the sharing of information on a patient’s emergency visit between chemotherapy and emergency nurses?	15	24.2	47	75.8
**Interdisciplinary Learning**				
7. Do you have any interdisciplinary learning opportunities regarding oral chemotherapy?	20	32.3	42	67.7
**System-based Approach for Detecting Prescription Errors**				
8. Is there any system-based approach for detecting prescription errors?	27	43.5	35	56.5

n=62.

### Patient-based survey

Mean number of chemotherapy patients per day was 23.9 (SD 20.22).

Demographic and clinical characteristics of the 249 chemotherapy patients are presented in Table 4. A combination of intravenous and oral chemotherapy was administered to 222 patients (89.2%). More than half of patients, both male and female, were over 65 years of age. Gastrointestinal cancer was by far the most common (69.9%), and 75.4% of treatments were for recurrences.

**Table 4:** Patient characteristics

**Gender**	**n**	**%**
Male	142	57.0
Female	107	43.0
**Age**		
<65	115	46.2
≧65	133	53.4
Unknown	1	0.4
**Type of Cancer**		
Breast cancer	30	12.0
Gastrointestinal cancer	174	69.9
Lung cancer	16	6.4
Others (e.g., hematological, uterine and ovary cancer)	26	10.4
Unknown	3	1.2
**Purpose of Treatment**		
Primary treatment	61	24.5
Recurrence treatment	188	75.5
**Type of Treatment**		
Combination of oral and intravenous chemotherapy	222	89.2
Only oral chemotherapy	22	8.8
Unknown	5	2.0

n=249.

Table 5 shows the responses of nurses to the questions on nursing practices related to medication adherence for the 249 patients on oral chemotherapy. Questions on side effects, discussions about barriers to achieving balance between treatment and daily life activities, and medication management were all significantly related to the question about unused medicines.

**Table 5:** Patient-based survey: unused medicines and other adherence-related questions (n = 249)

			**Asking about unused medicines**		**P value**
			**Yes**	**No**	
Do you talk about side effects with the patient?	Yes	214 (85.9%)	108 (98.2%)	106 (76.3%)	<0.001
	No	35 (14.1%)	2 (1.8%)	33 (23.3%)	
Do you talk about barriers to balance between treatment and daily life activities with the patient?	Yes	181 (72.7%)	101 (91.8%)	80 (57.6%)	<0.001
	No	68 (27.3%)	9 (13.2%)	59 (42.4%)	
Do you think that this patient manages his/her medication (oral chemotherapy) at home?	Yes	230 (92.4%)	105 (95.5%)	125 (89.9%)	0.148
	No	19 (7.6%)	5 (4.5%)	14 (10.2%)	
Do you think that monitoring medication management (oral chemotherapy) for this patient is your role?	Yes	202 (81.1%)	104 (94.5%)	98 (70.5%)	<0.001
	No	47 (18.9%)	6 (5.5%)	41 (29.5%)	

Fisher’s exact test.

### Factors predicting adherence-related nursing practices

Given that the question on unused medicines could be used as a kind of proxy for adherence-related nursing practices, a multivariable logistic regression model was created. The results of the logistic regression analysis are shown in Table 6. Nurses’ experience, education and qualifications, the combination of oral and intravenous treatment, patient orientation on oral chemotherapy, interdisciplinary learning, and having a system-based approach for detecting prescription errors remained significant. The factors explained 40.3% of the variance in the model.

**Table 6:** Factors predicting adherence-related nursing practices by logistic regression

	**Odds ratio (95% CI)**		**p-value**
*Nurse factor*			
Years of experience	1	-	.008
6-10 (years)	4.209	(1.240 - 14.284)	.021
<10	1.028	(0.320 - 3.300)	.963
Education University	.016	(0.003 - 0.073)	<.001
Oncology certified nurse	5.786	(2.337 - 14.324)	<.001
*Patient factor*			
Oral + IV combination therapy	.240	(0.070 - 0.828)	.024
*System factor*			
Patient orientation	3.348	(1.633 - 6.861)	.001
Interdisciplinary learning	2.997	(1.469 - 6.114)	.003
System-based approach for detecting prescription errors	3.671	(1.865 - 7.226)	<.001

*Note.* CI = confidence interval.

Variables entered: years of experience, education, certification; age, type of cancer, regimen; patient orientation, telephone follow-up, emergency contact, patient consultation and education, and education for emergency nurse, interdisciplinary, and system-based approach for detecting prescription errors.

## Discussion

This is the first report on current nursing practices for patients receiving oral chemotherapy in Japan. There was great variation in practices across the nation. The survey results demonstrated that a majority of the cancer centers and large hospitals in Japan are responding to the emerging needs of oral chemotherapy in conventional care systems, with only a minority incorporating systems specific to oral chemotherapy. Adherence-related nursing practices were associated with the nurse’s background, the type of treatment, and healthcare system-related factors.

First, nurses should be aware of their role in monitoring the medication management of patients receiving oral chemotherapy. In the present study, nearly 20% of nurses were not aware of their monitoring role. Although nurses educate patients about intravenous chemotherapy, few are involved in oral chemotherapy [5]. As understanding the clinical importance of oral chemotherapy is reported to encourage adherence in 90% of patients [3], patient should be made well aware of both the benefits of medication and the risks of non-adherence [14]. Patient education is the cornerstone of successful oral chemotherapy [8,15]. A feasibility pilot study of new medication adherence interventions suggests that nurses have an important role to play in education, monitoring, and follow-up [21]. A strong patient-nurse relationship is fundamental to individualized education, leading to successful management of and adherence to treatment regimens [4].

The nurses were unlikely to ask how patients understood the therapy and how they managed medication at home, and very few nurses asked whether patients were confident in medication management. Patients may not be confident in medication management, but nurses do not know how they feel about isolation. At least, nurses should ask patients whether they are taking their medication as prescribed. Information the nurse could gain from this simple question is worth to ask. The answer implies patient adherence to medication. Recently, nurses’ contact with patients is decreasing [18]. It is because oral chemotherapy is often administered outside of controlled settings [7], and patients usually receive medication information directly from the oncologist or pharmacist without nursing support [16]. Regular contact with a single nurse helps promote patients’ trust [22], and facilitates identification of concerns about treatment and daily life activities. The patient-nurse relationship and collaboration of the healthcare team are essential components of patient adherence [23]. A proactive attitude on the part of the nurse is a key in caring for patients on oral chemotherapy.

Healthcare-system related factors were predictors of adherence-related nursing practices in this study. Such factors included patient orientation with an overview of oral chemotherapy presented by a nurse, interdisciplinary learning opportunities, and a system-based approach for detecting prescription errors. As oral chemotherapy becomes more common, more patients will require education. Patient orientation for oral chemotherapy should be systematically implemented for every patient who initiates this type of therapy. Patient education about the disease, treatment, and symptom management is the role of the oncology nurse [24], and staffing for orientation is important to ensure patients receive the necessary education.

Currently, healthcare providers have little ability to directly manage oral chemotherapy care [25]. There is great value in creating an efficient and effective interdisciplinary team among healthcare providers in cancer care [26]. Interdisciplinary learning opportunities remind team members of their roles, integrate professional knowledge, and facilitate collective work [27].

Regarding prescription errors, intravenous chemotherapy is commonly double-checked or triple-checked by multiple staff in controlled settings. The safety issues in oral chemotherapy are just important as those in intravenous chemotherapy [2], however, the safe delivery of oral chemotherapy may depend on the competency of those dispensing the medication [7]. Including a set routine in an organization’s safety infrastructure can raise the awareness of healthcare providers on safety and monitoring of administration of oral anticancer agents. Institutional safety infrastructure is critical for favourable clinical outcomes [15].

In the present study, nurses asked about adherence to medication regimens more frequently when patients were on oral chemotherapy alone, rather than on combined intravenous and oral chemotherapy. This may have been in part because nurses must focus on strict standards in administering intravenous chemotherapy, and therefore might not have the time to ask about oral medication. Adherence is not only an issue for patients new to oral chemotherapy, but also for those with refilled prescriptions [28]. As shown in the results of the present study, nurses are less likely to monitor medications of patients with refilled prescriptions. Cancer patients tend to undergo long-term treatment, and therefore the concept of follow-up, keeping track of the condition of cancer patients, is important. Shared accountability among multiple providers in cancer care makes individual accountability blur. In addition to individual commitment to care, major improvement requires coherent strategies of the organization.

There are a number of limitations to the present study. Systematic sampling is vulnerable to periodicities which might lead to bias. Very few hospitals approved the survey because of strict regulations on patient data. We confirmed that all hospitals received invitations to participate in the survey, but could not reach the person in charge at all the hospitals we approached. Thus, low response rate may have produced a bias if the replies from respondents were not representative of the national sample. The present study relied on the nurses’ response to the questionnaires. We did not measure actual patient adherence to medication regimens. This survey focused on chemotherapy clinics; outpatient and primary care settings were not included. Therefore, the results of the survey do not reflect the overall picture of oral chemotherapy treatment. The results of the logistic regression suggested that nurses with higher education were unlikely to ask about unused medicines, which is difficult to explain; however, there were only 6 nurses who had educational backgrounds higher than university. This small number might have produced the unexpected result.

## Conclusions

A multicenter cross-sectional survey revealed that adherence-related nursing practices were associated with the nurse’s background, type of treatment, and healthcare system-related factors, including patient orientation on oral chemotherapy, interdisciplinary learning, and having a system-based approach for detecting prescription errors. With the increase of oral chemotherapy, a new model of patient care is required. A more systematic approach should be developed to ensure patients receive safe and effective oral chemotherapy, while nurses should play significant roles in patient education and monitoring.

Further studies are needed to develop an organized system of care in which nurses proactively support patients in managing safe and effective oral chemotherapy and improve medication adherence with coordination among healthcare providers.

## Competing interests

All authors declare that they have no competing interest.

## Authors’ contributions

HK and KY^1^ designed the study, developed the methodology, collected the data, performed the analysis, and wrote the manuscript. KY^2^ provided his perspective regarding the study design and performed statistical analyses as a clinical epidemiologist. All authors read and approved the final manuscript.
